# Statement of Retraction: Clinical significance of serum MicroRNA-203 in patients with acute myeloid leukemia

**DOI:** 10.1080/21655979.2020.1800321

**Published:** 2020-08-04

We, the Editor, Publisher and author, have retracted the following article from *Bioengineered*:

Yingmeng Guo, Clinical significance of serum MicroRNA-203 in patients with acute myeloid leukemia, *Bioengineered*, 2019;10(1): 345-352, DOI:10.1080/21655979.2019.1652490

The above article has been retracted as, subsequent to publication, significant overlap in text, study and figures has been identified with the following earlier published article:

Zhuanzhen Zheng, Gong Rong, Guoxia Li, Fanggang Ren, Yanping Ma, Diagnostic and prognostic significance of serum miR-203 in patients with acute myeloid leukemia, *International Journal of Clinical and Experimental Pathology*, 2019;12(5):1548-1556, DOI 1936-2625/IJCEP0092247

We have been informed in our decision-making by our policy on publishing ethics and integrity and the COPE guidelines on retractions.

The retracted article will remain online to maintain the scholarly record, but it will be digitally watermarked on each page as ‘Retracted’.

